# Varieties

**Published:** 1862-08

**Authors:** 


### VARIETIES.

COOL off slowly after all forms of exercise by avoiding drafts of air, or sitting at an open door or window.

THAT excellent and most reliable paper, the *Scientific American*, of New-York City, $2 a year, advises the ladies, when they wish to wash fine and elegant colors, to boil some bran in rain-water, and use the liquid cold. Nothing, it is said, can equal it for cleaning cloth and for revivifying effects upon colors.

FORCING FOOD, that is, eating when you are not hungry, is a wicked waste, is fighting against nature, and puts you below the level of a brute, for brutes never go against their instincts.

A SMALL pinch of gunpowder given to a chicken with the gapes, will effect a cure in from one to three hours' time, and leave poor chick healthy.

ICE-WATER in summer is a great enemy to the teeth, an impairer of digestion and a promoter of dyspepsia, although it is comfortable to take when very thirsty.

POTATOES, the finest, mealiest, and most healthful, will sink in very salt water; the soft and waxy swim.

THE editor of the New-York *Observer*, an amateur and practical fruit-grower, declares in the most positive terms that "Two applications of whale-oil soap-mixture in a dry season (or more, if rainy) will protect the fruit from the Curculio." We presume the ingredients should be in a proportion to make it of a consistence which will admit of sprinkling or syringing.

FRUITS are healthful. A lady in Gainstown bought eight acres of worn-out stony land at forty dollars an acre, and set it out in an orchard at an expense of two hundred dollars. She cropped it every year, cleared two hundred dollars a year, and at the end of six years after the purchase refused twenty-five hundred dollars for the field.

E. LAKE, of Topsfield, Mass., gathered from one acre, of Baldwin russet apples two hundred barrels, at four dollars, besides one and a half tons of marrow-squashes and one hundred heads of cabbages, one of which weighed twenty-seven pounds.

To protect trees from rabbits, mice, etc., rub the bark from the ground to the hight of eighteen inches with blood or raw, bloody meat, (fresh liver is best,) late in the fall.

A WELL-BRED family is kind, polite, and cheerful, at meal time, in every word, gesture, and look. The brutal, the low-born, and inherently vicious scarcely fail once in a week to sit down with a complaint, a growl, or a contemptible whine. Even a pig wags its tail in satisfaction while its nose is in the slop-trough. It is left to human brutes alone to eat in thankless, ungracious fault-finding.

LAWNS must be swept frequently, and mown once a week until July, and gradually less often until October. Now, ladies can mow lawns themselves, for Boyd's Brush Lawn-Mower is made small enough for a lady to guide or draw, and no scythe can equal the machine-work, for the grass is cut as even as velvet; but it must be done regularly, and not be allowed to get ahead.

